# Evaluating the Effectiveness of Cellulose-Based Surfactants in Expandable Graphite Wood Coatings

**DOI:** 10.3390/polym16192832

**Published:** 2024-10-07

**Authors:** Tereza Jurczyková, Elena Kmeťová, František Kačík, Martin Lexa, Daniel Dědič

**Affiliations:** 1 Department of Wood Processing and Biomaterials, Faculty of Forestry and Wood Sciences, Czech University of Life Sciences Prague, Kamýcká 129, 16000 Prague, Czech Republic; lexa@fld.czu.cz (M.L.); dan.dedic@seznam.cz (D.D.); 2 Department of Fire Protection, Faculty of Wood Sciences and Technology, Technical University in Zvolen, 96001 Zvolen, Slovakia; xkmetovae@is.tuzvo.sk; 3 Department of Chemistry and Chemical Technologies, Faculty of Wood Sciences and Technology, Technical University in Zvolen, 96001 Zvolen, Slovakia; kacik@is.tuzvo.sk

**Keywords:** fire protection, fire-retardant, nanocellulose, cellulose ethers, timber, scanning electron microscopy, relative mass loss, burning rate

## Abstract

This study deals with the design of modern environmentally friendly and non-toxic flame retardants based on expandable graphite 25 K + 180 (EG) modified by cellulose ethers (Lovose TS 20, Tylose MH 300, Klucel H) and nanocellulose (CNC) that are biocompatible with wood and, therefore, are a prerequisite for an effective surfactant for connecting EG to wood. The effectiveness of the formulations and surfactants was verified using a radiant heat source test. The cohesion of the coating to the wood surface and the cohesion of the expanded graphite layer were also assessed. The fire efficiency of the surfactants varied greatly. Still, in combination with EG, they were all able to provide sufficient protection—the total relative mass loss was, in all cases, in the range of 7.38–7.83% (for untreated wood it was 88.67 ± 1.33%), and the maximum relative burning rate decreased tenfold compared to untreated wood, i.e., to 0.04–0.05%·s^−1^. Good results were achieved using Klucel H + EG and CNC + EG formulations. Compared to Klucel H, CNC provides significantly better cohesion of the expanded layer, but its high price increases the cost of the fireproof coating.

## 1. Introduction

Wood is undoubtedly the most advantageous and readily available natural raw material. However, the susceptibility of wood products to fire is an essential challenge in the wood industry. The fire behavior of wood is a very complex phenomenon due to different components in various quantities and their specific and independent reactions to fire [1].

It is well known that wood comprises three main polymeric components: cellulose, hemicelluloses, and lignin, and is supplemented by a small proportion of extractives [2]. Although the chemical and anatomical composition of wood varies according to individual wood species, climatic conditions of growth, macro-, and micro-location, etc., the processes during exposure to fire are well-known and predictable at a practical level [3]: At approximately 100 °C, dehydration of cell walls occurs, followed by a thermal softening process between 180 and about 300 °C, and finally thermal degradation of the individual constituents occurs [4,5]. Hemicelluloses decompose at a lower temperature (ca. 180–350 °C), while cellulose degrades at a relatively higher temperature (ca. 275–350 °C). Cellulose depolymerization starts with the breakage of a link in the carbon ring and further crosslinking of the polymer chain to produce the end products of pyrolysis [2,6]. Due to its heavily crosslinked structure, the relatively stable lignin decomposes at temperatures of around 250–500 °C [7]. The main pyrolysates from wood are volatile gases, levoglucosan, and carbonaceous char [6,7,8].

In the literature [9,10,11,12,13], there are a lot of experimental studies concerning the fire hazard of wood, which demonstrate the influence of various factors on the fire hazard indices (species of wood, conditions, duration of operation, humidity, fire intensity, etc.). In these studies, the pyrolysis and thermal oxidative degradation of wood are examined, thermal and physical characteristics are determined, and carbonization rates are obtained for various temperature modes. These data can be used to evaluate the fire resistance of wooden structures.

The flammability of wood can be minimized or delayed by fire-retardant treatments. Wood flame retardants are effective compounds that lower the temperature of the pyrolysis reaction, delay ignition, or reduce the heat release rate. At the same time, they also increase the amount of charred material and reduce or dilute the amount of volatile flammable gases [14,15]. Within the chemical protection of wood, there is a contact of wood mass with inorganic or organic substances of different chemical structures [16,17,18], which counteract the activity of the degradation factors. Wood preservatives, fire retardants, fungicides, insecticides, etc., are usually a combination of one or more active ingredients, other auxiliary substances, and solvents [19]. Woodfire retardants are typically applied by high-pressure impregnation, chemical modification, surface coatings, integration of active compounds into the glue system, or through the addition of wood-based materials during manufacturing [1,20].

The history of the use of fire retardants in wood can be traced back to early civilizations, such as ancient Rome, when wood was impregnated with vinegar and lime to build fire-resistant warships and housing [21]. After the Great Fire in the first century in Rome, wood was soaked in salt water [22]. Research on fire retardants for wood continued in the following centuries. The earliest references found relating to fire retardants are the British patent no. 551, which was issued to Obadiah Wyld in 1735, and the scientific article published by Gay-Lussac in 1821 [23]. In the 1980s, guanidine phosphates were synthesized and used as fire retardants for wood at Northeast Forestry University [24]. Since the 1990s, more research efforts have been focused on wood fire retardants containing phosphorus, nitrogen, and boron compounds or mixtures of these [21]. They are among the most important and can be classified into different categories, such as inorganic, organic, resin-based, reactive, or other types, according to their chemical nature and application characteristics. However, only a few fire retardants display the performance required for use in wood treatment [25].

The requirements for fire retardants for wood have expanded from an initial focus on fire retardancy to encompass other factors such as smoke inhibition, environmental impact, preservative performance, and economic aspects. They should possess nontoxicity, durability, high potency, maintain good dimensional stability, and be relatively cheap and wieldy [26,27]. The stipulations in relevant regulations and standards for wood fire retardants have become stricter, making the design and development of new wood fire retardants more difficult [21]. New strategies [28,29] such as chemoenzymatic modifications, plasma impregnation, and nanocomposites are still in the research stages and are yet to be commercialized. Another interesting approach is in situ reactions inside the wood to prevent the leaching of fire-retardant chemicals. Fire-retardant alternatives may be based on, for instance, DNA, proteins, saccharides, or phytic acid [30].

The goal in this study is to design modern flame retardants for wood, which focus on preparing green and bio-derived flame retardants that are non-toxic and environmentally friendly and meet most of the above requirements for effective modern wood fire retardants. This new formulation uses, in addition, expandable graphite (EG) from a natural source, modified by cellulose in the form of cellulose derivatives and nanocellulose, which are biocompatible with wood and, therefore, are a prerequisite for an effective surfactant for connecting graphite to wood. This contribution follows on from our previous studies, in which the suitability and effectiveness of the use of EG in wood retarders was sufficiently verified [31,32,33], and in which we used water glass as a surface-active substance. It also follows on from and develops the pilot study [34] on the use of nanocellulose as a surfactant for EG. As is clear from previous studies, nanocellulose and water glass themselves have retarding effects, which are improved by adding EG. In this study, we focus on other biocompatible surfactants from natural sources that would be appropriate for using EG on wood.

EG has many uses and, as an intumescent flame retardant, improves the flame-retardant properties of many materials and industries [35,36]. EG is a modified graphite that has a layered structure with interlayer space. Its production occurs through the interaction of crystalline graphite flakes with concentrated sulfuric acid and other potent oxidizing agents, e.g., nitric acid or potassium permanganate. As a flame retardant, its dilatability and thermal stability parameters are essential. When the specific starting temperature for each type is exceeded, the EG begins to expand, creating a porous carbonized layer with high thermal insulation capabilities. This is achieved by fixing the intercalant between the graphene layers, which, upon exposure to the required temperature, turns into a gas phase consisting of water vapor and sulfur dioxide, which results in an approximately 100-fold increase in the volume of the intercalant. The pressure generated by the volume increase forces the individual graphite layers to separate, creating an insulating char layer forming a physical barrier between the protected material and the flame. This layer maintains excellent heat resistance and is very porous, allowing air to flow and cool the fire environment [37,38]. From our previous research as well as from the studies of other authors [34,39,40,41], it follows that the properties of EG (particle size, expansion ratio, decomposition temperature) influence the retardation effect and the properties of the polymeric surfactant affect the slowing down of wood decomposition to a lesser extent. In addition to its fire-retardant properties, EG has sufficient electrical conductivity, as well as high strength and stiffness [42,43].

Cellulose, as the most widespread biopolymer on the earth, is widely found not only in plants and wood but also in various organisms, such as fungi, algae, bacteria, and certain marine invertebrates, such as tunicates [44,45,46]. It is a linear polymer that links D-glucose with 1,4-β-glycosidic bonds with many hydroxyl groups exposed in its structure. Through intramolecular and intermolecular hydrogen bonds and van der Waals interactions, cellulose is observed as microfibrils composed of amorphous and crystalline domains in varying proportions, depending on its source [47]. Moreover, the hydroxyl groups can be modified and chemically crosslinked to form a more stable network structure [48,49]. Compared to synthetic surfactants in various wood protective coatings, cellulose-based surfactants are renewable biological resources and display several advantages, such as low price, nontoxicity, good biocompatibility, relatively low environmental pollution, recyclability, outstanding mechanical characteristics, microporous architecture, chemical stability, and excellent hydrophilic characteristics [50].

Cellulose derivatives are cellulose whose reactive hydroxyl groups are chemically modified by various reactions such as esterification and etherification [51], giving derivatives including cellulose esters (e.g., cellulose acetate), cellulose ethers (e.g., carboxymethyl cellulose, methyl cellulose, ethyl cellulose), cellulose sulfate, and cellulose nitrate, etc. [52,53]. Cellulose ethers used in this study are made by the reaction of cellulose with aqueous sodium hydroxide and then with an alkyl halide, such as methyl or ethyl chloride [54]. These substances are capable of increasing the viscosity of aqueous media. They are soluble in water and are not toxic. The viscosifying ability of cellulose ethers is primarily controlled by its molecular weight, the chemical substituents attached to it, and the conformational characteristics of the polymer chain [55]. Cellulose ethers have played a significant role in various applications, from construction products, ceramics, paper, textiles, adhesives, and paints to foods, cosmetics, oil recovery, agriculture, and pharmaceuticals [56,57]. For construction products, cellulose ethers act as thickeners, binders, film formers, and water-retention agents. They also function as suspension aids, surfactants, lubricants, protective colloids, and emulsifiers. In addition, aqueous solutions of certain cellulose ethers thermally gel, a unique property that plays a crucial role in many other applications. Cellulose ethers have fair to good resistance to dry heat.

Nanocellulose can be mainly categorized into cellulose nanocrystals (CNCs), cellulose nanofibers (CNFs), and bacterial cellulose (BC). These nanomaterials have received significant interest due to their mechanical, optical, chemical, and rheological properties. CNCs that were used for our research, primarily obtained from naturally occurring cellulose fibers by acid hydrolysis, are biodegradable and renewable. Hence, they are a sustainable and environmentally friendly material [47]. CNCs are rigid rod-shaped particles with a 5–50 nm diameter and a length of 100–500 nm [58]. The utilization of CNCs for various applications (e.g., biomedical engineering, material sciences, electronics, green catalysis, biosensing, etc. [59,60,61,62]) can be of two broad types: one type involves the use of functionalized or nonfunctionalized as-synthesized CNC, and the other one consists of the use of polymer nanocomposites wherein CNC acts as a reinforcing agent. Due to their extensive surface area and the possibility of acquiring a negative charge during hydrolysis, large quantities of substances can be bound to their surface with optimal control of dosing [63].

The versatility of expandable graphite material has been attested by the manufacturer by the various polymeric coating systems in which it was incorporated. Nearly all existing premier coating solutions have used this graphite-based intumescent coating, including epoxies, latex, silicones, siloxanes, bitumen mixes, polyurethanes, and many hybrid structures [64]. Charmeau et al. [65] published that the presence of surfactants in latex coatings has been shown to strongly influence the adhesion properties. The majority of surfactants used in coating formulations are standard anionic and non-ionic amphiphiles, such as fatty alcohol sulfates, alkylaryl sulfonates, and alcohol ethoxylates. Cationic and amphoteric surfactants are rarely used. In recent years, polymerizable surfactants have become of interest. By using surfactants that become covalently attached to the latex particle, many of the problems encountered with stability and the adhesion of dried film in different outdoor conditions can be avoided or at least minimized [66,67,68]. Eksted [69] mentioned that surfactants in alkyde resin coatings hurt the ability of the coating to prevent water ingress, which is most probably due to the hydrophilic character of these substances. The presence of these substances alters the moisture sorption characteristics of wood. Considering that these substances, common in waterborne wood coatings, may be mobilized during and after film formation, and accumulate at the coating/substrate interface, there is a high probability that these substances change the moisture sorption characteristics of the wood substrate unfavorably and create unexpected dynamic moisture conditions at the coating/wood interface [70,71]. In the case of our previous research testing EG coatings with water glass [32], it is therefore necessary to bear in mind that water glass has a strong alkaline reaction in water conditions. Cellulose ethers, investigated in this study as surfactants, provide many benefits: lubrication, increased crack-crack resistance, anti-slip, increased adhesiveness, and extended open time. They are high-purity, non-ionic polymers designed for use as thickening and film-forming agents, protective colloids, suspending and emulsifying agents, binders, and stabilizers [72]. Therefore, cellulose ethers compete with synthetic water-soluble polymers (polyvinyl alcohol, polyuretane associative thickeners, polyacrylates) and other natural water-soluble polymers (xanthan gum, carrageenan, locust bean gum), although they are also soluble in water. The striking behavior of cellulose ethers in water is based on the hydrophobic effect, which causes an increased order of the polymer surrounding water molecules [73]. Depending on their type (polarity and surface functionality) and quantity, it is therefore possible to expect a certain water sensitivity of formulated water-based wood coatings and a negative influence on the coating’s adhesion under humid conditions (wet adhesion). Water resistance, or water sorption and dissolution, is therefore another important criterion that the proposed coatings should meet, and therefore this property will be verified immediately in the next phase of testing.

In this study, cellulose-based surfactants in combination with EG were tested and evaluated as natural biocompatible fire-retardant coatings for spruce wood. The evaluation of fire resistance during the test with a radiant heat source was carried out by determining parameters such as the relative mass loss, relative burning rate, and surface temperature of the treated samples. At the same time, the adhesion of the coating to the wood surface was assessed using scanning electron microscopy (SEM), and the cohesion of the graphite layer was tested after expansion. Based on the experimental data, it is possible to describe the advantages and disadvantages of newly prepared fire-protective coatings for wood and recommend the most appropriate of them. 

## 2. Materials and Methods

### 2.1. Materials

#### 2.1.1. Wood

Samples of Norway spruce wood (*Picea abies* (L.) H. Karst) originating from a 120-year-old stand at 800 m above sea level without anatomical or other defects were selected for this experiment. For the radiant heat source test, the samples were sawn from the dried trunk part of the tree to the dimensions 10 × 40 × 50 mm^3^, and their surface was treated with planing. All the samples were air-conditioned for 21 days at 21 ± 2 °C and 65 ± 5% relative humidity to reach an equilibrium moisture content of approximately 12%.

#### 2.1.2. Expandable Graphite (EG)

Based on our previous results [34], EG from the company Epinikon a.s. (Vodňany, Czech Republic) was selected, namely type 25 K + 180, which has average values of all specified particle properties and, compared to other EGs, showed, together with the product GG 200-100 N from the NeoGraf company (Lakewood, OH, USA), the best test results for the response of treated wood surface to the exposure to a certain degree of heat flux. EG 25 K + 180 is characterized by a starting temperature of 180–220 °C, a pH value 5–9, a particle size of at least 80% of the content > 180 μm, a carbon content of at least 95%, and an expansion volume of 250 mL/g.

#### 2.1.3. Cellulose-Based Surfactants

Four cellulose-based substances were tested for possible use as surfactants from natural sources biocompatible with wood. All the substances were compared with each other in terms of the effective connection of EG Epinikon 25 K + 180 to the wood surface, the ability to ensure cohesive EG even after its expansion when exposed to high temperature, and in terms of their fire retardation properties. These surfactants were nanocrystaline cellulose and three cellulose ethers—Lovose TS 20, Tylose MH 300, and Klucel H. The characteristics of the individual substances are provided below.

##### Cellulose Ethers

Cellulose derivatives come from the same raw material: pure cellulose from wood or cotton. This pure cellulose is made alkaline by reaction with NaOH. After this preparation, various ethers can be prepared using various etherification agents. Different reagents are used depending on the ether desired [74]. Cellulose ethers are characterized by the fact that, after drying, they form firm, flexible, colorless films with microbial resistance and health safety. They are reversible and have a constant pH value. A certain hygroscopicity must be taken into account [75].

- Lovose TS 20 (LOV)

Producer: Lovochemie a.s. (Lovosice, Czech Republic); Trade name: for a mixture of sodium carboxymethylcellulose (min. 64%), sodium carbonate (<3%), and sodium hydroxide (<2%). It is an odorless white to pale ocher substance, which is soluble in water to form a gel, which, upon further dilution below 10%, dissolves into a viscous solution (the viscosity of a 1% solution at 20 °C is 10–20 mPa·s and the pH is 10–11.5). The substance’s safety data sheet states that type TS 20 presents a low risk for spreading fire, compared to type T 20. LOV is used in many industries due to its properties of anti-redeposition (detergents), for stabilizers (dispersion and emulsion), thickeners (painting paints), adhesives and binders (finishing agents, paper sizing, wallpaper adhesives, etc.). In addition to the paper, textile, and fat industries, it is also used in the construction and oil industries [75,76].

- Tylose MH 300 (TYL)

Producer: Kremer Pigmente GmbH & Co. KG, (Aichstetten, Germany); Trade name: methylhydroxyethylcellulose (MHEC). The number 300 indicates the viscosity of a 2% solution and is proportional to the size of the macromolecule. The reagents for the preparation of MHEC are methyl chloride and ethylene oxide. The degree of substitution varies between 1.5 and 2.0. TYL solutions and other cellulose ethers are prepared in concentrations of up to 10% when they are still spreadable. The reaction of the TYL solution is neutral or slightly alkaline and can be adjusted by adding acids or bases. Both aqueous and aqueous ethanol solutions can be prepared. It is used as a thickener, glue, paper impregnation agent, finishing and separating agent, paint binder, etc. [76]. Aqueous solutions with a number higher than 1000 are used for gluing [77].

- Klucel H (KLU)

Producer: Kremer Pigmente GmbH & Co. KG, (Aichstetten, Germany); Trade name: hydroxypropylcellulose (HPC). It is a cellulose ether prepared by reacting propylene oxide with the hydroxyl groups of cellulose. It is a white, odorless, and tasteless substance. The viscosity of a 1% solution of Klucel H at 20 °C is 1500–3000 mPa·s, relative molecular weight M_w_ ≈ 1,150,000. Although its adhesive and consolidating properties are not outstanding, KLU has penetrated practice thanks to other important properties—it combines thermoplasticity with surface activity with the thickening and stabilizing properties of different, purely water-soluble cellulose derivatives. It also can only dissolve in cold water (at temperatures above 40 °C, KLU is already insoluble and therefore waterproof). KLU is also soluble in anhydrous alcohols and in acetone. A certain amount of non-polar solvents (e.g., toluene) can be added to these solutions. These properties make it possible to use KLU as an auxiliary or alternative adhesive [77]. Klucel H is a highly pure substance (also used in microbiology and pharmacy), as is reflected in its price. 

##### Nanocrystaline Cellulose (CNC)

CNC fulfills the function of a surfactant in the proposed fire-retardant formulation—it ensures the adhesion of the coating with the surface of wood and graphite, even after it expands when exposed to high temperature [34]. At the same time, it has a certain fire retarding effect [78,79]. Used CNC-Com-Regular (Producer: Cellulose Lab, Fredericton, NB, Canada) is transparent and odorless and is found in slurry form with a solid content of 6% suspension in water. It is characterized by hydrophilic surface properties and has hydroxyl and sulfonic surface groups. Its fiber dimensions are 5–20 nm wide and 100–250 nm long.

### 2.2. Methods

#### 2.2.1. Recipe Design of EG Fire-Retardant Coatings with Individual Surfactants

In our previous research [34], which dealt with the study of CNC and its possible use in the form of a surface-active substance for connecting EG to the surface of wood, an optimal formula was found. The intention was to saturate the EG surfactant solution as much as possible, but only to the extent that the prepared mixture has good application properties. At the same time, the spreadability of the formulation, uniformity of the coating, flowability on vertical surfaces, drying time, and adhesion to wood after drying were evaluated. For this follow-up study, the composition of the formulation was 2 g of dry matter CNC in 100 mL of a 5% (wt/wt) NaOH alkaline solution with 80 g EG.

In the case of cellulose derivatives (LOV, TYL, KLU), the aim was to preserve the preparation procedure (except for the alkaline solution, which only makes sense in the case of CNC [80]) and the mixture ratios. However, when preparing a 2% (wt/wt) solution of KLU in water, a very viscous mixture was formed in which the graphite particles could not be well dispersed, and the consistency of the formulation would not be suitable for spreading. For this reason, the surfactant concentration was reduced to 1% (wt/wt), even for LOV and TYL, for a better comparison within the group of cellulose ethers. In the preparation of 1% (wt/wt) aqueous solutions of cellulose ethers, intensive stirring was required and complete dissolution was achieved by moderate heating within 30 min. When the same consistency of the EG-surfactant mixture was used for the cellulose derivatives as for the CNC mixture, the formulation was very pasty, application to wood was difficult, and the coating was uneven. Therefore, formulations were tested with a reduced amount of EG, always by 5% (wt/wt), until the stage when the coating met all the requirements for a good application, non-flowing, homogeneity, and drying time. The choice of 1 g of dry matter cellulose ether (LOV/TYL/KLU) dissolved in 100 mL of distilled water with the subsequent addition of 60 g of EG proved to be the best formulation for all cellulose ethers. In the case of KLU, the recipe of 1 g KLU in 100 mL of ethanol (according to the recommendations of the technical sheet of the product) with 60 g EG was additionally tested. Despite the good solubility of the surfactant itself in alcohol, the formulation did not work—the graphite particles in the mixture settled at the bottom, the paint spread poorly and ran off vertical surfaces, and above all, there was insufficient cohesion of the paint with the wood. Four best-rated test mixtures of surfactants with EG are listed in Table 1.

#### 2.2.2. Application on Wood

**Figure 1:**
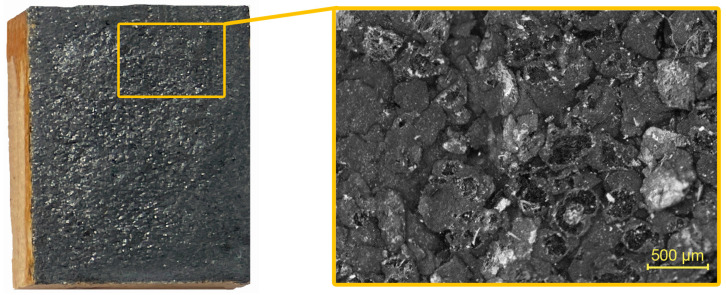
A coating of the surfactant + EG formulation (in this case specifically CNC) on a surface of spruce wood and a detailed view of the EG 25 K + 180 particles under 40× magnification.

The prepared formulations were uniformly applied with a brush with synthetic fibers on the surface of the samples 40 × 50 mm (Figure 1) in such an amount as to achieve the same amount of EG 1.00 ± 0.05 g. Upon drying, the exfoliated graphite lamellae by cellulose-based surfactants were reassembled, forming a continuous adherent film, regardless of the substrate roughness. Furthermore, the wood test samples were painted only with individual surfactants (LOV/TYL/KLU/CNC) to determine the heat resistance of the surfactant itself. Surfactants were applied in the same amount corresponding to the proportion of surfactant in the coating with EG on the prepared samples. A control of the applied amount of formulations and surfactants was carried out by weighing the sample on analytical scales with an accuracy of 2 decimal places. All the coatings prepared in this way had a thickness of 442–495 μm, as subsequently determined by image analysis obtained using a Nikon Eclipse Ni polarizing microscope (Nikon, Tokyo, Japan) at 40× magnification and 0.85 μm/px resolution. The structure and quality of the coatings were also observed and evaluated with a polarizing microscope at a 40× magnification. The coating surfaces of all the samples treated with EG 25 K + 180 with different cellulose-based surfactants did not differ in appearance (distribution of graphite flakes, holes, or cracks in the structure, etc.) at this magnification, and the selected surfactants had the same function in this aspect of coating formation. The character is thus dominantly indicated by graphite. A typical surface of the newly designed coatings is shown in Figure 1, specifically the CNC + EG formulation.

A total of 9 test series of 10 samples each were prepared for the radiant heat source test: 4 series treated with formulations with EG differing in surfactant (LOV + EG, TYL + EG, KLU + EG, CNC + EG), 4 series treated with individual types of surfactants (LOV, TYL, KLU, CNC), and 1 series of untreated wood samples (REF).

#### 2.2.3. Radiant Heat Source Test

The radiant heat source test is a non-standard method used in model combustion tests. Said method makes it possible to measure the mass loss of the material under radiant heat loading. The experiment consists of exposing the test specimens to the effect of a thermal infrared heater with a different heat output for a certain time, at various distances from the surface of the radiating body, and its evaluation criteria are the relative mass loss (δ_m_(τ)), ignition time of samples (τ_i_), relative burning rate (ν_r_), and temperature course on the top of the samples.

In this experiment, a ceramic thermal infrared heater (Ceramicx, Cork, Ireland) with a constant electric power of 1000 W and Radwag PS 3500.R2 electronic scales (Radwag, Radom, Poland) were used. The duration of action was 600 s and the distance of the samples from the surface of the heater was 40 mm. The mass loss was recorded every 10 s (using the R-Lab software, version 2018.4.11). Any ignition of the samples was visually checked with a time record if this phenomenon occurred. Subsequently, the relative mass loss and relative burning rate of wood from the measured values were calculated. With the thermal camera Fluke RSE600 (Fluke Corporation, Everett, WA, USA), images were taken during the test using the Smart View R&D software IRSoft2 (version 7.0) at a selected point—on the top of the samples in 1-s intervals [34].

The cohesion of the expanded layer was tested by simply rotating the sample by 180° after finishing the test with a radiant heat source. The efficiency was evaluated based on the weight loss of the expanded layer after rotating the sample, i.e., how much of the EG fell off. The individual test series were compared with each other.

#### 2.2.4. Microscopy Analysis

Expanded graphite layers were imaged using a Nikon SMZ1270 stereomicroscope (Nikon, Tokyo, Japan) equipped with a Plan Apo 1x objective with a 70-mm working distance and a Nikon DS-Fi3 digital camera (Nikon, Tokyo, Japan). The NIS-Elements AR 5.42.05 software was used for scanning the samples. Focused composite images were compiled for some of the samples that did not allow for perfect surface focusing.

The morphology of fire-protective coatings, particle dispersion, and adhesion to the wood surface were evaluated using scanning electron microscopy (SEM). The treated wood samples were cut into thin cross-sections and subsequently gold-coated using a laboratory coater Q150R ES (Quorum Technologies Ltd., Lewes, UK). The microscopic analysis was carried out using scanning electron microscope MIRA 3 (Tescan Orsay Holding, a.s., Brno, Czech Republic). A secondary electron detector was used and the acceleration voltage was 12 kV.

## 3. Results and Discussion

### 3.1. Relative Mass Loss

**Figure 2:**
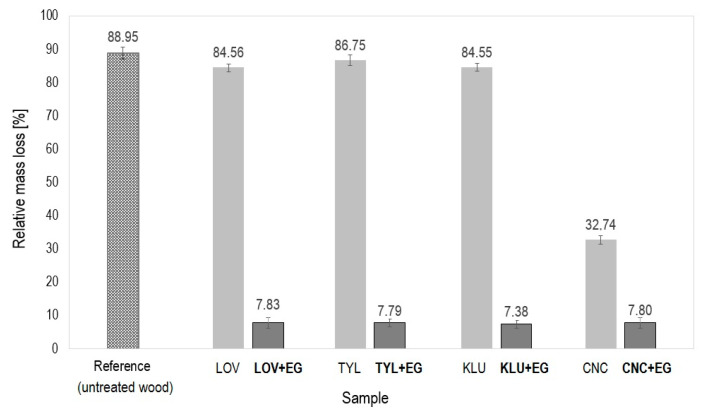
Relative mass losses after the radiant heat source test for the untreated wood sample (REF), samples treated only with surfactants (LOV, TYL, KLU, CNC), and samples treated with the appropriate surfactant in combination with EG (LOV + EG, TYL + EG, KLU + EG, CNC + EG). Abbrev.: LOV—Lovose TS 20, TYL—Tylose MH 300, KLU—Klucel H, CNC—Cellulose nanocrystals CNC-Com-Regular, EG—Eexpandable graphite 25 K + 180.

The graph in Figure 2 presents the calculated total relative mass loss values of all the examined samples at the end of the test with a radiant heat source, which were treated with surfactants and formulations composed of surfactant and EG. The average value of the relative weight loss of the REF sample (88.67 ± 1.33%) is shown for comparison.

The results for the samples treated with surfactants show that the cellulose ethers themselves, i.e., LOV, TYL, and KLU, have only a minimal effect on the thermal protection of wood, and compared to untreated wood, an improvement in relative weight loss of a maximum of 4.4% was achieved. LOV and KLU have an almost identical effect, with TYL being the least effective against thermal action, i.e., reducing the relative weight loss compared to the REF sample by only 2.2%. In contrast, very good results were achieved in the case of CNC. This confirms the recently published reports on the improved flame retardancy performance of commercial polymers in the presence of surface-modified cellulose nanocrystals or cellulose nanoparticle hybrids with other fire retardants [81,82,83]. In this case, unlike the other tested surfactants, CNC prevented the samples from burning for the entire test duration. Nanocellulose alone ensures more than twice the protection of the material against the effect of fire compared to untreated samples, i.e., the relative weight loss in the radiant heat source test is approximately 56% lower than the REF sample.

The darker bars in the graph in Figure 2 show the relative weight losses for samples treated with formulations consisting of the respective surfactants and EG. Very good results were achieved for all the samples treated with the proposed formulations, regardless of the surfactant. The best, i.e., the lowest, values of relative weight loss (7.38 ± 0.72%) were achieved when using the KLU + EG formulation, followed by the TYL + EG, CNC + EG, and LOV + EG formulations, whose values differ negligibly and range from 7.79 to 7.83%. These results prove that EG has a dominant retarding function in the fire-retardant coating, and the potential fire-retardant properties of surfactants are overshadowed.

When compared to the results of the previous study [32], where the same type of EG 25 K + 180 was used, and a concentrated aqueous solution of water glass—WG (sodium silicate) was chosen as the surfactant, a slight improvement can be observed in the case of cellulose-based surfactants, since the overall relative weight loss using the WG + EG formulation is approximately 8.08 ± 0.78%. On the other hand, when treating a spruce wood sample with only WG, the lowest observed decrease in relative weight was only 19.45 ± 0.32%. However, it should be noted that the samples treated with WG + EG coating were still additionally treated with WG on the surface. If the same additional surface treatment was carried out in the case of our samples, the retardation effects could theoretically be stronger, the relative weight losses lower, and the differences with the compared WG + EG formulations more significant in favor of the cellulose derivatives.

**Figure 3:**
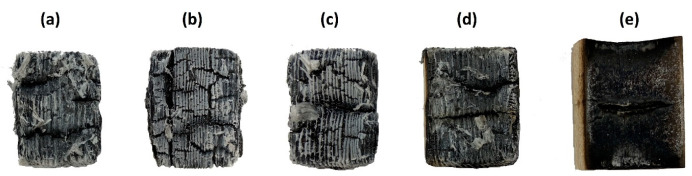
Untreated wood (REF) (**a**) and wood samples treated only with surfactants, i.e., with (**b**) LOV, (**c**) TYL, (**d**) KLU, and (**e**) CNC, after 600 s of exposure to a radiant heat source.

Figure 3 shows the appearance of the samples treated with individual cellulose-based surfactants, from which the total relative weight loss was determined after the test with a radiant heat source. The appearance of the samples treated with proposed EG coatings after the radiant heat source test is presented further on in the Section 3.3 Evaluation of Expanded Layer Cohesion.

### 3.2. Evolution of Relative Mass Loss and Relative Burning Rate in Time and Sample Surface Temperature Course during a Radiant Heat Source Exposure

**Figure 4:**
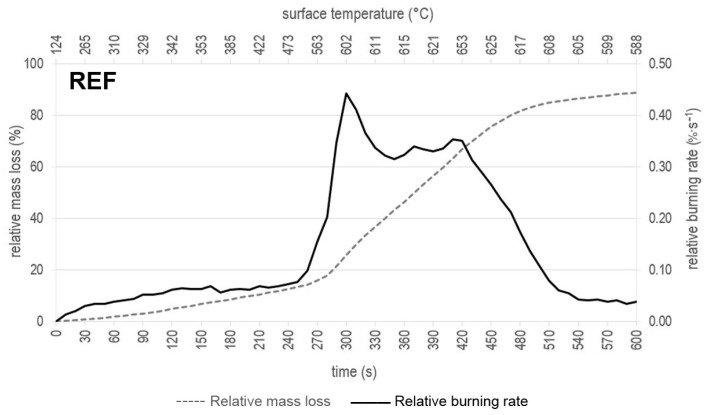
Record of evaluated parameters during a test with a radiant heat source for the untreated spruce wood sample (REF).

**Figure 5:**
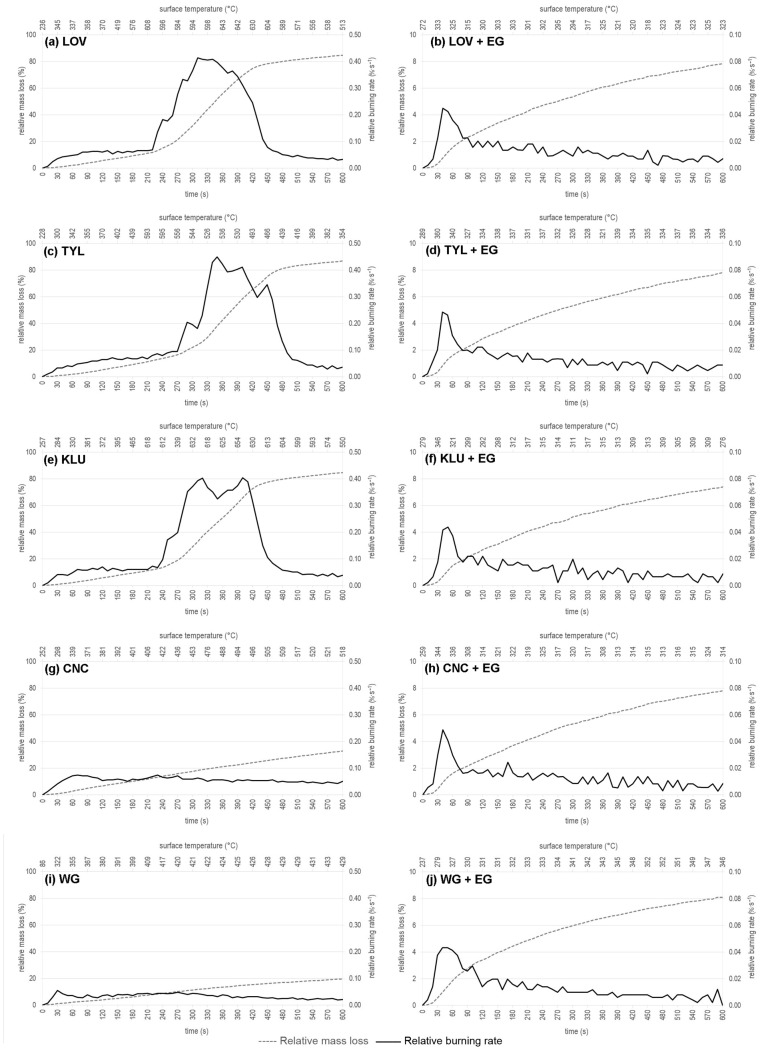
Record of evaluated parameters during a test with a radiant heat source for investigated wood samples treated only with cellulose derivates: (**a**) Lovose TS 20, (**c**) Tylose MH 300, (**e**) Klucel H (**g**) CNC; with formulations composed of surfactants based on cellulose and expandable graphite: (**b**) Lovose TS 20 + EG 25 K + 180, (**d**) Tylose MH 300 + EG 25 K + 180, (**f**) Klucel H + EG 25 K + 180, (**h**) CNC + EG 25 K + 180; and with (**i**) Water glass and (**j**) its formulation with EG 25 K + 180 for comparison. Abbrev.: LOV—Lovose TS 20, TYL—Tylose MH 300, KLU—Klucel H, CNC—Cellulose nanocrystals CNC-Com-Regular, WG—Water glass, EG—Expandable graphite 25 K + 180.

All the experimental results of monitoring the relative mass loss, relative burning rate, and time to ignition for the REF sample and samples treated with only surfactants as well as formulations composed of surfactant and EG 25 K + 180 are presented in Figure 4 and Figure 5. Samples treated with WG and the WG + EG 25 K + 180 formulation from our previous study [32] are also included for comparison in Figure 5.

When evaluating the development of the relative weight loss of the samples over time (δ_m_(τ)) of the test 0–600 s, it can be seen from Figure 4 that the greatest relative weight loss of the REF sample occurs between 270 s and 480 s. A similar trend can be observed in the case of samples treated with cellulose ethers in Figure 5a,c,e. A significant decrease in δ_m_(τ) also occurs from 270 s (with LOV from 240 s); however, stabilization of δ_m_(τ) appears a little earlier than in the case of REF—already at 430 s with LOV, 440 s with KLU, and 470 s with TYL. For samples treated only with CNC (Figure 5g) and WG (Figure 5i), surfactants δ_m_(τ) during the test gradually increase to a total value of 32.7 ± 0.54% for CNC and 19.45 ± 0.32% for WG. All the samples treated with formulations combining surfactant and EG (Figure 5b,d,f,h), including WSG (Figure 5j) from the previous study [32], show a very similar trend of a gradual increase in δ_m_(τ) to the final range of values of 7.38–7.83%, as already mentioned above, and respectively 8.08 ± 0.78% for WSG.

If we compare the samples based on the development of relative burning rate (ν_r_) and surface temperature (T_s_), the highest values were measured not only for the REF sample but also for more or less all cellulose ethers:, i.e., for REF ν_r_ = 0.44%·s^−1^ at T_s_ ≈ 602 °C, for LOV ν_r_ = 0.41%·s^−1^ at T_s_ ≈ 594 °C, for TYL ν_r_ = 0.45%·s^−1^ at T_s_ ≈ 530 °C, for KLU ν_r_ = 0.40%·s^−1^ at T_s_ ≈ 631 °C. Treatment with TYL significantly reduces the surface temperature of the sample within this evaluated group, especially in the second half of the test with a radiant heat source—a reduction of up to 33%. The highest surface temperatures were about 654 °C and were recorded for the untreated sample and the sample treated with KLU in times of 420 s and 390 s, respectively. The value intervals when ν_r_ > 0.1%·s^−1^ for this evaluated series of samples are as follows: REF 260–500 °C, LOV 230–440 °C, TYL 270–490 °C, and KLU 240–450 °C. During glucose-based polymer decomposition, physical dehydration happens by releasing the stored moisture, followed by thermal condensation of hydroxyl groups in higher temperatures. Subsequently, aromatic rings (e.g., furan) are formed at around 400 °C due to water liberation and other ether segment formations. These aromatic rings later participate in carbonization and char formation [84,85]. It is worth mentioning that the thermal stability of cellulose ethers is highly dependent on their composition, structure, chemical modification, and preparation methods.

**Figure 6:**
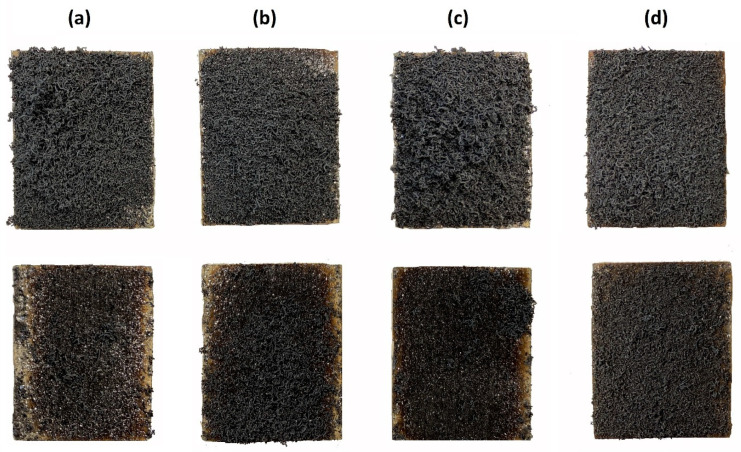
Wood samples treated with coatings composed of (**a**) Lovose TS 20 + EG 25 K + 180, (**b**) Tylose MH 300 + EG 25 K + 180, (**c**) Klucel H + EG 25 K + 180, (**d**) CNC + EG 25 K + 180 after EG expansion (upper line) and after expanded layer cohesion test (lower line).

Up to a 6-fold reduction in ν_r_ compared to REF and, thus, the first evaluated series of samples treated with cellulose ethers occurs for wood treated with CNC (ν_r_ = 0.07%·s^−1^ at T_s_ ≈ 339 °C) and WG (ν_r_ = 0.05%·s^−1^ at T_s_ ≈ 322 °C). T_s_ also decreases by up to 45% in both cases at the maximum value of ν_r_. A significant reduction in ν_r_ (up to 10-fold compared to REF) occurs for samples treated with the proposed formulations composed of surfactant and EG. The trends of the ν_r_ development during the test are almost identical and again the partial effect of the surfactant itself is overshadowed. The highest values of ν_r_ for all the tested samples treated with the formulations, including the comparison with the WG + EG formulation, are 0.04–0.05%·s^−1^ at a time of 40 s in the case of using cellulose derivatives in the formulation and 30 s when using WG. The ν_r_ parameter decreases more slowly during the time in the case of samples treated with WG + EG compared to the other samples tested in the current experiment. The surface temperatures of these samples at the maximum value of ν_r_ are the lowest in this experiment for wood treated with KLU + EG (T_s_ ≈ 318 °C). A slight increase is observed for other samples: T_s_ ≈ 332 °C for LOV + EG, T_s_ ≈ 343 °C for CNC + EG, and T_s_ ≈ 347 °C for TYL + EG. Over time, the temperatures drop slightly and then maintain a constant course in the range of 330–310 °C until the end of the test, depending on the sample type. Only in the case of KLU + EG does the T_s_ of the sample decrease below 300 °C at the end of the test, even to 276 °C. This decrease corresponds to a decrease of approx. 20% compared to T_s_ coupled with maximum ν_r_. We assume that KLU allows for easier expansion of the EG, thereby creating a thicker protective layer after heat exposure (Figure 6). As a result, the T_s_ of the sample is lower compared to the other tested surfactants in combination with EG. When comparing these results with the samples treated with WG + EG, the T_s_ of the sample at the maximum value of ν_r_ is comparable to those treated with the LOV + EG formulation, i.e., T_s_ ≈ 329 °C. Compared to samples with cellulose derivatives used in the formulation, the trend of T_s_ development of samples treated with WG + EG is the opposite (increases up to 7%) and is undesirable.

### 3.3. Evaluation of Expanded Layer Cohesion

The structure of the inorganic expansion layer has an important influence on fire resistance. The structure of the inorganic expansion layer isolated the wood from the heat and flame, achieving the purpose of fire resistance [86]. The appearance of the investigated samples with an expanded fire-protection layer is shown in Figure 6 in the upper line, as well as the appearance of the same samples after the cohesion test, when the samples were rotated by 180 °C so that any insufficiently cohesive part of the expanded layer could fall off. The weight of the samples before and after this test was recorded and a simple evaluation was performed on this basis.

From the point of view of the cohesion of the expanded layer of EG 25 K + 180 with the expansion volume of 250 mL/g, the formulation with CNC surfactant appears to be the best (after the rotation test, there was a loss of material from the expanded layer <5 wt.%). On the contrary, insufficient cohesion was confirmed for samples using LOV and TYL surfactants, when approximately 16.4 ± 1.21 wt.% or 18.9 ± 0.98 wt.% graphite after expansion fell off. In the case of the formulation with KLU, the weight loss was 9.6 ± 1.14 wt.%. Cohesion in mixtures of tested surfactants with EG depends on the structure and functional groups of individual polymers. As a result of the formation of hydrogen and van der Waals bonds between the polymer and EG [87,88,89,90,91,92,93,94], an adhesive film is formed which has different strength properties. In the case of CNC, thanks to the many free hydroxyl groups, the film is very strong and the layer is therefore the most cohesive. Surfactants LOV and TYL, on the other hand, probably create fewer intermolecular hydrogen bonds and the cohesion of the coating prepared from them is lower after graphite expansion. Generally, improving the compactness, strength, and stiffness of the layer after expansion could be done in the future by additional treatment of the coating with a surfactant solution itself or by surface color treatment.

**Figure 7:**
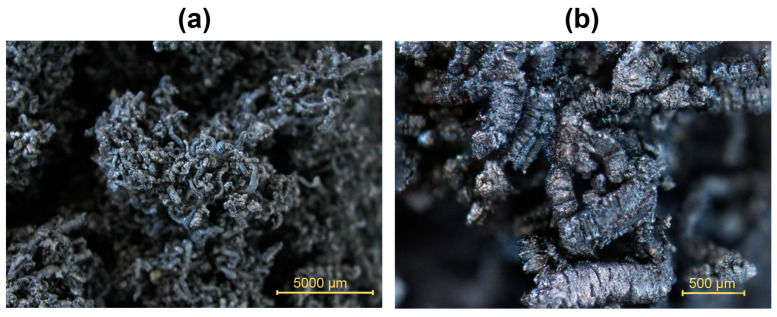
Expanded layer of 25 K + 180 graphite in formulation with CNC. Magnification (**a**) 6.3× and (**b**) 40×.

Microscopy images with focus composite adjustment created during observation with a stereomicroscope of investigated EG coatings with 25 K + 180 graphite particles after expansion under (a) 6.3× and (b) 40× magnification are presented in Figure 7. The character of the expanded layers is identical at the chosen magnification for all the samples regardless of the surfactant used, and it is given by the EG used with a particle size > 180 μm and an expansion volume of 250 mL/g. The expanded layer in this form has an important influence on fire resistance. 

### 3.4. Evaluation of Coating Adhesion to Wood by SEM

SEM provided additional helpful information about the surfactant-bonded EG layer with the wood surface.

**Figure 8:**
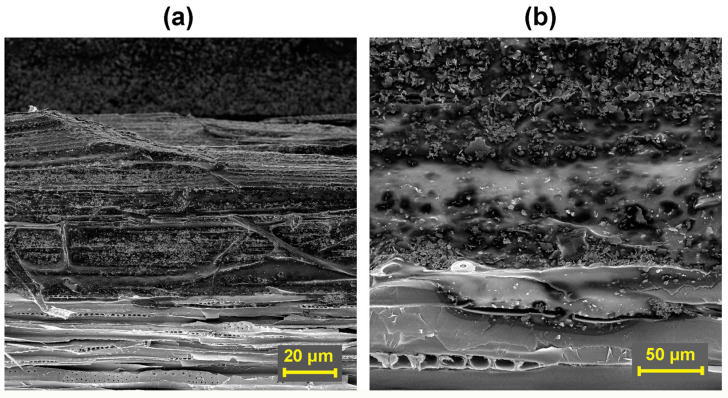
Spruce wood sample treated (on a radial section) only with Klucel H surfactant: (**a**) Evaluation of wood tangential section, magnified 200× (**b**) Detail of the layer penetrated by the surfactant, magnified 1000×.

**Figure 9:**
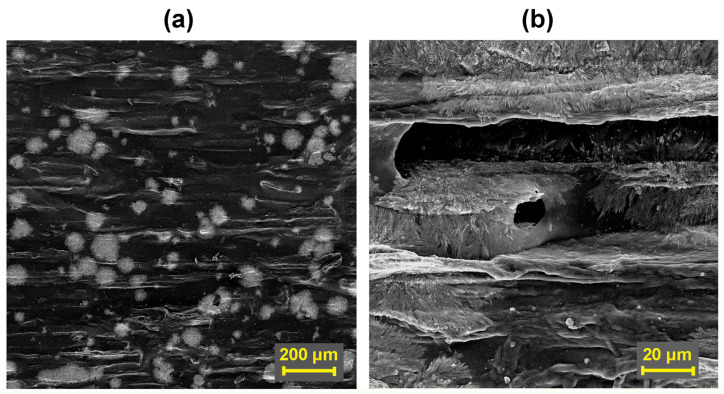
Spruce wood sample treated (on a radial section) only with CNC surfactant: (**a**) Treated wood surface, magnified 200× (**b**) CNC crystals on a radial section around the colon, magnified 2000×.

On tangential sections of spruce wood samples treated with individual surfactants, it was possible to observe the depth of penetration of the surfactant itself into the wood structure (Figure 8a), which ranged from approximately 0.7 mm (in the case of KLU) to 1.1 mm (CNC). With TYL and LOV, the penetration of the surfactant to a depth of approximately 0.8–0.9 mm was recorded. The surfactant in the wood structure has the character of an adhesive and can ensure sufficient connection through a film-forming coating after drying. The adhesive mixture also includes small clusters of undissolved cellulose ethers (Figure 8b) or recrystallized CNC (Figure 9a,b), which can also be observed on a radial section or surface of a wood sample treated with this surfactant.

**Figure 10:**
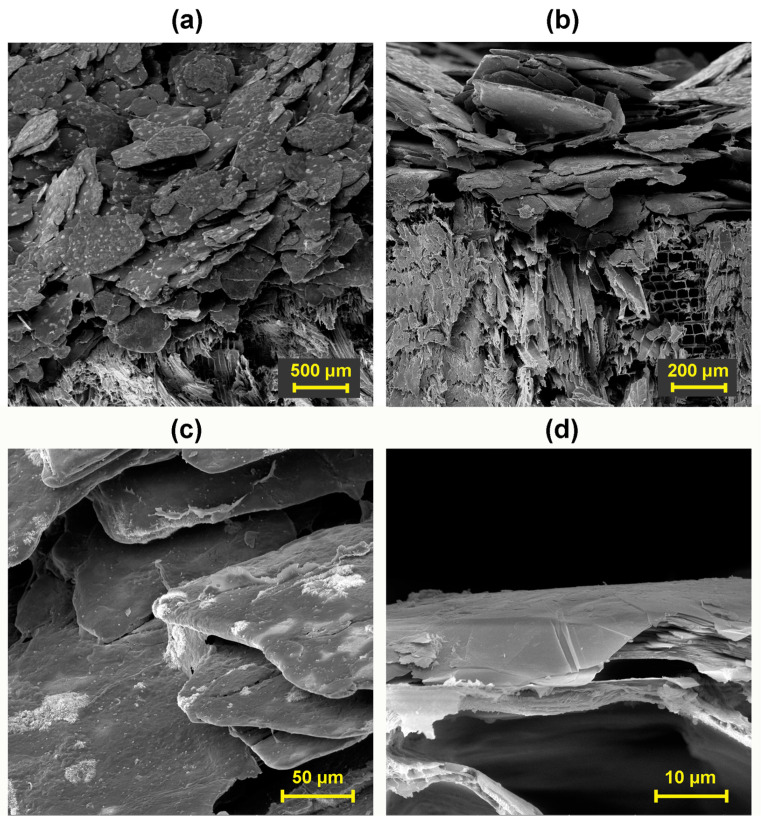
Fire-protection layer composed of CNC + EG 25 K + 180: (**a**) On wood surface, magnified 100×, (**b**) Coating layer profile, magnified 200×, (**c**) Detail of EG particles connected by CNC, magnified 1000×, (**d**) Intercalated EG layers on the graphite-wood interface and connection through CNC adhesive film 5000×.

In Figure 10a, it is possible to observe mainly particles of EG 25 K + 180 deposited in a layer with a thickness of approximately 460 μm connected by the CNC surfactant on the surface of the wood in its tangential section. Likewise, as in the study by Santos et. al. [80], the SEM micrograph of the finished fire-resistant coating shows a flat layer of irregular particles that are predominantly aligned along the surface plane. CNC is also seen in the coating side view lying on the lamellae flat surfaces as well as in between them. CNC occupies only a fraction of the graphitic particle surfaces. At a higher magnification in Figure 10b, the exfoliated layers of EG and their arrangement in the graphite fireproof coating are already clearly visible. Graphite exfoliation has been described in the literature using various dispersing solvents, surfactants, polymers, oxidizing to graphene oxide, high pressure, or another mechanochemical action [80]. In Figure 10c,d, the binding function of the surfactant can be seen even within the graphite layer between individual EG particles. The surfactant creates a uniform adhesive film on the surface of the wood and on the EG particles, which ensures the adhesion of the coating to the wood and the cohesion of the graphite layer itself. The adhesion mechanism of all the tested surfactants is similar. However, the degree of binding interactions and efficiency varies depending on the type of surfactant, as confirmed by the cohesion test.

**Figure 11:**
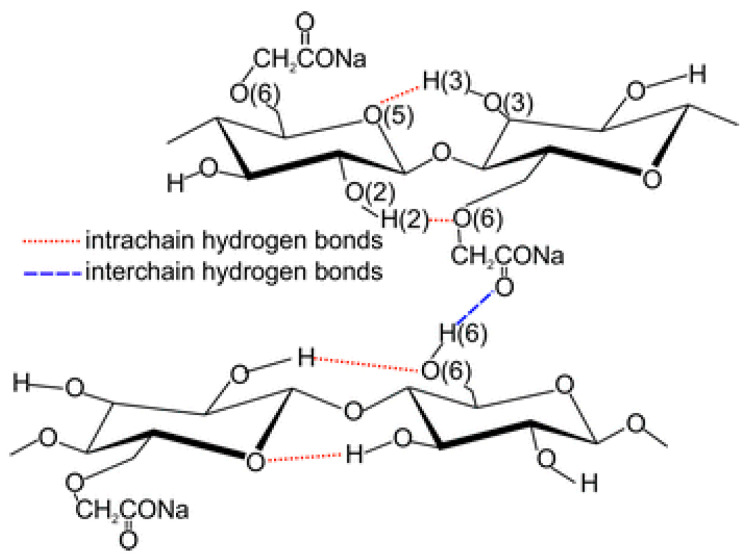
The scheme of hydrogen bonds in Lovose TS 20 aqueous solutions [87].

The cellulose ethers used in this study and CNC have many polar functional groups leading to crosslinking networks (Figure 11) due to hydrogen bonds and van der Waals forces [87,88,89,90]. These functional groups can form intermolecular hydrogen bonds with the main components of wood, which creates a solid film on the wood surface [91,92,93]. EG also contains polar functional groups, especially hydroxyl ones [94]. Therefore, it is likely that EG not only gets into the surfactant film mechanically but also creates mutual bonds with its functional groups.

## 4. Conclusions

This study brings new possibilities for solving the problem of connecting expandable graphite in fire-resistant coatings with wood surfaces with the help of natural, biocompatible substances from renewable sources based on cellulose, which introduces almost no other chemicals into the wood-expandable graphite system.

The results confirmed the suitability of using CNC in a formulation with EG, which by itself has a significant retarding effect on wood and even surpasses WG in certain evaluated parameters. The disadvantage of using CNC is undoubtedly its high cost. In case of transfer into practice, it is therefore necessary to find a local supplier or to consider the production of CNC from waste biomass.

An important finding is that the influence of the possible fire-retardant properties of the surfactant itself in the formulas is negligible, and all this effectiveness is attributed to EG. The importance of the surfactant primarily lies in its ability to ensure maximum adhesion of the EG formulation to the wood surface and, at the same time, sufficient cohesion of the graphite particles after expansion.

Following nanocellulose, three other cellulose-based substances (Tylose MH 300, Lovose TS 20, Klucel H) were tested as a possible replacement for CNC in the formulation, mainly due to better availability and lower formulation prices. From the point of view of the best values of relative mass loss, it would be possible to recommend KLU as the most suitable surfactant. Moreover, the price of KLU could be further reduced by using its more affordable variant with lower viscosity, assuming the same surfactant effects (Klucel M with M_w_ ≈ 850,000 or even Klucel G with M_w_ ≈ 370,000), which would certainly work much better when preparing the formulation than with the rather viscous type H (M_w_ ≈ 1,150,000). Compared to LOV, TYL, and CNC, KLU also provides the lowest maximum relative burning rate at the lowest surface temperature, which has at the same time the most pronounced downward trend. From the group of evaluated cellulose ethers, KLU shows the best adhesion of the formulation to wood and the cohesion of the layer itself after expansion. However, it is still almost twice as weak as CNC. The improvement in cohesion could be solved in the future by the above-mentioned method applied to the sample with water glass when the coatings of the formulations were finally coated with the surfactant itself for stabilization and better cohesion. It would also be appropriate to test the preparation of cellulose ether in an alkaline aqueous solution, as in the case of CNC, which could contribute to better exfoliation of graphite and, at the same time, a higher binding capacity.

## Figures and Tables

**Table 1:** Prepared formulations of expandable graphite 25 K + 180 (Epinikon) with all investigated cellulose-based surfactants (LOV, TYL, KLU, CNC).

Surfactant Type/ Mixture Components	Lovose TS 20	Tylose MH 300	Klucel H	CNC
Surfactant dry matter (g)	1	1	1	2
Solvent type	Distilled water	Distilled water	Distilled water	5% (wt/wt) NaOH
Solvent volume (mL)	100	100	100	100
EG content in formulation (g)	60	60	60	80

## Data Availability

Additional data will be made available upon reasonable request to the authors.
